# Projecting productivity losses for cancer-related mortality 2011 – 2030

**DOI:** 10.1186/s12885-016-2854-4

**Published:** 2016-10-18

**Authors:** Alison Pearce, Cathy Bradley, Paul Hanly, Ciaran O’Neill, Audrey Alforque Thomas, Michal Molcho, Linda Sharp

**Affiliations:** 1 National Cancer Registry Ireland, Building 6800 Cork Airport Business Park, Kinsale Rd, Cork, Ireland; 2 Virginia Commonwealth University, Richmond, VA 23284 USA; 3 National College of Ireland, Mayor Street, IFSC, Dublin 1, Ireland; 4 National University of Ireland, University Road, Galway, Ireland; 5 Newcastle University, Richardson Road, Newcastle upon Tyne, NE2 4AX UK; 6 Centre for Health Economics Research and Evaluation, University of Technology Sydney, Sydney, Australia

**Keywords:** Neoplasms, Cost of illness, Work, Employment, Labor force, Premature Mortality, Health care economics, Productivity, Household activities, Human Capital Approach

## Abstract

**Background:**

When individuals stop working due to cancer this represents a loss to society – the loss of productivity. The aim of this analysis was to estimate productivity losses associated with premature mortality from all adult cancers and from the 20 highest mortality adult cancers in Ireland in 2011, and project these losses until 2030.

**Methods:**

An incidence-based method was used to estimate the cost of cancer deaths between 2011 and 2030 using the Human Capital Approach. National data were used for cancer, population and economic inputs. Both paid work and unpaid household activities were included. Sensitivity analyses estimated the impact of assumptions around future cancer mortality rates, retirement ages, value of unpaid work, wage growth and discounting.

**Results:**

The 233,000 projected deaths from all invasive cancers in Ireland between 2011 and 2030 will result in lost productivity valued at €73 billion; €13 billion in paid work and €60 billion in household activities. These losses represent approximately 1.4 % of Ireland’s GDP annually. The most costly cancers are lung (€14.4 billion), colorectal and breast cancer (€8.3 billion each). However, when viewed as productivity losses per cancer death, testis (€364,000 per death), cervix (€155,000 per death) and brain cancer (€136,000 per death) are most costly because they affect working age individuals. An annual 1 % reduction in mortality reduces productivity losses due to all invasive cancers by €8.5 billion over 20 years.

**Conclusions:**

Society incurs substantial losses in productivity as a result of cancer-related mortality, particularly when household production is included. These estimates provide valuable evidence to inform resource allocation decisions in cancer prevention and control.

## Background

Over 40 % of those diagnosed with cancer in Europe are of working age [1] and this proportion is increasing due to a growing emphasis on early diagnosis, improved treatment outcomes, and rising retirement ages [2]. When individuals exit the workforce temporarily or permanently due to cancer, this represents a loss of productivity for society. Similarly, productivity is also lost when someone is unable to do unpaid production tasks, such as housework, caring and volunteering. These losses can be valued in monetary terms.

Together with measures of burden such as incidence and mortality, estimates of cancer-related productivity loss provide valuable evidence that can inform population-based resource allocation decisions in cancer prevention and control [3]. The patterns of cancer in society are changing due to population ageing, early detection and improved treatment [4] suggesting that the cancer burden, irrespective of how it is measured, will change in coming years. By projecting productivity losses into the future, decision makers can account for these changes when allocating resources.

Cancer mortality-related productivity losses have been projected for the United States (US) from 2000–2020, and estimated that annual productivity lost due to cancer mortality would rise from USD$116 billion in 2000 to USD$148 in 2020 [5]. In Europe however, only single year estimates are available [3, 6, 7]. Premature cancer-related mortality in Ireland has been estimated to cost €510 million annually [3]. While useful, these estimates do not provide sufficient information to estimate the potential economic savings of implementing cancer interventions that impact future populations. In addition, only two of these previous studies [3, 5] included unpaid productivity, which approximately doubled the estimates of lost productivity.

The aim of this study was to estimate national productivity losses associated with premature mortality from all adult cancers combined and from the 20 highest mortality adult cancers in Ireland in 2011, and project these losses until 2030.

## Methods

### Setting and approach

Ireland has a population of 4.6 million people [8], and there are approximately 18,500 invasive cancers diagnosed in Ireland each year, and 8300 cancer deaths [9]. Over 45 % of those diagnosed with cancer in Ireland are of working age (<65 years). Over the last 20 years, cancer incidence has increased by approximately 3 % per year due to population growth and aging [9], while cancer mortality rates have declined by about 1 % per year [9]. Ireland has a relatively young population, with approximately 66 % aged 15 to 64 years [10] and although participation rates are somewhat below those in Europe, a rising proportion of the population are in the workforce [11].

An incidence-based method was used to estimate the cost of cancer deaths between 2011 and 2030. The methods were based on those of Bradley et al. [5] and used the Human Capital Approach, which measures lost productivity as the time the working life is shortened due to illness, valued at the market wage (or an equivalent proxy). Both paid work (paid production) and unpaid household activities (household production) were included, consistent with methods recommended for the societal perspective [12].

### Data sources

Age specific cancer mortality data was provided by the Central Statistics Office (CSO) and annual age-specific cancer mortality rates by cancer site for all cancers combined and the 20 most common cancers in terms of deaths were calculated by the National Cancer Registry Ireland. The CSO also provided national data for population and economic inputs. These included: a) population projections (using historical trends data) made in 2013 for 2016 to 2030, which assume a constant fertility rate and slowing international immigration and emigration [13]; b) projections (using historical trends data) of life expectancy between 2009 and 2030 [13]; c) annual earnings in 2009, including basic earnings, bonuses and benefits in-kind by gender and 10 year age group and averaged across full- and part-time workers [14]; d) labour force participation and unemployment rates in 2011 by gender and broad age groups (15–19, 20–24, then 10 year age groups to 64) [15].

Time estimates for household production by gender were obtained from the Organisation for Economic Cooperation and Development (OECD) up to the age of 65 [16] and from the Time Use in Ireland Survey for those aged over 65 [17]. Both surveys included routine housework, care for household members, shopping, volunteering and household related travel as household production [16, 17], and the OECD also included caring for non-household members. A proxy market wage for all household production roles was assigned from the public service pay scales of the Health Service Executive (the public body in Ireland responsible for the provision of health and personal social services), with ‘home help’ selected because it is a generalist role that could be employed to complete any of the tasks included in our definition of household production [18]. The OECD project the average GDP growth rate from 2011 to 2030 as 2.1 % [19], which was used as a proxy for wage growth. A discount rate of 5 % was used, as recommended for Ireland [20].

### Calculations

The number of cancer deaths annually between 2011 and 2030 was projected, by gender and five-year age group, by applying the 2007 to 2011 average cancer-specific annual mortality rate to the population projections for each year. The years lost until retirement for each death were then calculated as the number of years from the mid-point of the age group to the effective retirement age (the average age of actual retirement, as opposed to the official pensionable age) in Ireland: 64.6 years for males and 62.6 years for females [21].

Projected earnings were calculated accounting for wage growth, unemployment rates and workforce participation over the life course. For example, a 59 year old woman who dies of cancer in 2020 had a base wage in 2020 of €21,384, based on the 2010 base wage for women aged 50–59 plus 10 years of wage growth at 1.9 % (€41,409), and adjusted for workforce participation (59.7 %) and unemployment (13.5 %) rates of 50–59 year old women in 2020. For each year between diagnosis (age 59) and retirement (age 62.6) a wage was calculated, accounting for wage growth (1.9 %) from the previous year, and changes in participation and unemployment rates based on the increasing age of the individual. For example, in 2021 the woman would be 60 and have a probability of participation and unemployment of 48.9 % and 14.9 % respectively, giving an average wage of €17,559.49; a 61 year old woman in 2022 would have another year of wage growth, 48.9 % participation and 14.9 % unemployment and an average wage of €17,893.12, and so on. The annual wages were then summed and adjusted for annual discounting.

Household production was estimated for all cancer deaths using a replacement cost approach - the hourly rate to hire home help (€14.73/h) was multiplied by the average number of hours spent in household production. These costs were calculated from the age of cancer death until life expectancy using the same approach described for wages.

Results are presented as total costs and costs per cancer death (by dividing the total cost by the number of cancer deaths in the relevant age and gender groups), and are given in 2011 Euros. All data management and analyses were conducted in SAS 9.3.

### Sensitivity analyses

Four scenarios were investigated in sensitivity analyses. The first assumed a 1 % reduction each year in the number of cancer deaths per 100,000 (spread equally across cancer types, age groups and genders), consistent with conservative estimates of the trend in Ireland since 1994 [4]. The second varied the age of retirement, as Ireland has relatively high rates of employment past pensionable age [22]. Paid productivity losses were calculated to life expectancy instead of effective retirement age, which allows the proportion of individuals in Ireland who continue workforce participation after the pensionable age of 65 (13.6 % of males and 5.1 % of females [15]) to be included. The third used the age and gender specific earnings to value unpaid household production, to take an opportunity cost approach instead of a replacement good approach. The fourth tested the impact of lower wage growth (0 %), higher wage growth (3.5 %), lower discounting (0 %) and higher discounting (6 %) on the estimates.

## Results

Deaths from all invasive adult cancers are projected to increase annually in Ireland, with a total of over 233,000 deaths between 2011 and 2030. The total value of productivity loss from these deaths is €73 billion - €13 billion in paid production and €60 billion in household production. Annual lost productivity rises from €2.3 billion in 2011 (€467 million paid production and €1.8 billion household production) to €5.4 billion in 2030 (€811 million paid production and €4.7 billion household production). Table 1 reports the paid production and household production losses by cancer site in total and per cancer death.

**Table 1:** Total losses due to cancer deaths 2011 – 2030, for all invasive cancers combined and by site (2011 Euros)

Cancer (ICD-10 code)	Projected number of deaths	Value of lost paid production (million €)	Value of lost household production (million €)	Value of lost paid production per death	Value of lost household production per death
C01-C14: mouth & pharynx	3691	€344	€954	€93,100	€258,363
C15: oesophagus	9928	€662	€2322	€66,653	€233,866
C16: stomach	9455	€603	€2265	€63,742	€239,541
C18-C21: colorectal	27,631	€1321	€6567	€47,818	€237,664
C22: liver	6641	€438	€1682	€65,998	€253,272
C25: pancreas	13,488	€733	€3355	€54,379	€248,753
C34: lung	48,922	€2403	€12,493	€49,116	€255,369
C43: melanoma skin	3698	€481	€1109	€129,992	€299,904
C44: non-melanoma skin	2297	€137	€492	€59,682	€214,112
C50: breast	18,308	€1255	€7194	€68,563	€392,941
C53: cervix	2200	€342	€1218	€155,238	€553,708
C54: corpus uteri	2104	€67	€706	€31,851	€335,495
C56: ovary	7150	€314	€2626	€43,859	€367,284
C61: prostate	17,012	€318	€2794	€18,667	€164,223
C62: testis	280	€102	€81	€364,187	€287,579
C64: kidney	5495	€413	€1374	€75,164	€250,066
C67: bladder	5929	€247	€1240	€41,734	€209,143
C70-C72: brain and CNS	6513	€886	€2164	€135,982	€332,348
C81-C85: lymphoma	7838	€528	€1989	€67,419	€253,744
C90: multiple myeloma	4380	€190	€1032	€43,319	€235,617
C91-C95: leukaemia	6693	€454	€1691	€67,773	€252,662
Other invasive cancers not specified	25,558	€1373	€6282	€53,704	€245,775
C00-C96: all invasive cancers	233,168	€12,685	€60,427	€54,403	€259,155

The most costly cancers are lung (€14.4 billion), colorectal and breast cancer (€8.3 billion each). Lung cancer results in the highest paid productivity losses (€2.4 billion), which is almost twice as costly as the next cancers – colorectal and breast cancers (€1.3 billion each). The same cancers are most costly for household production: lung (€12 billion), breast and colorectal (€7 billion each).

When viewed as paid production lost per cancer death, the most costly cancers are testis (€364,000 per death), cervix (€155,000 per death) and brain (€136,000 per death). In contrast, for household production losses per death the most costly cancers are cervix (€554,000 per death), breast (€393,000 per death), and ovary (€367,000 per death).

**Fig. 1:**
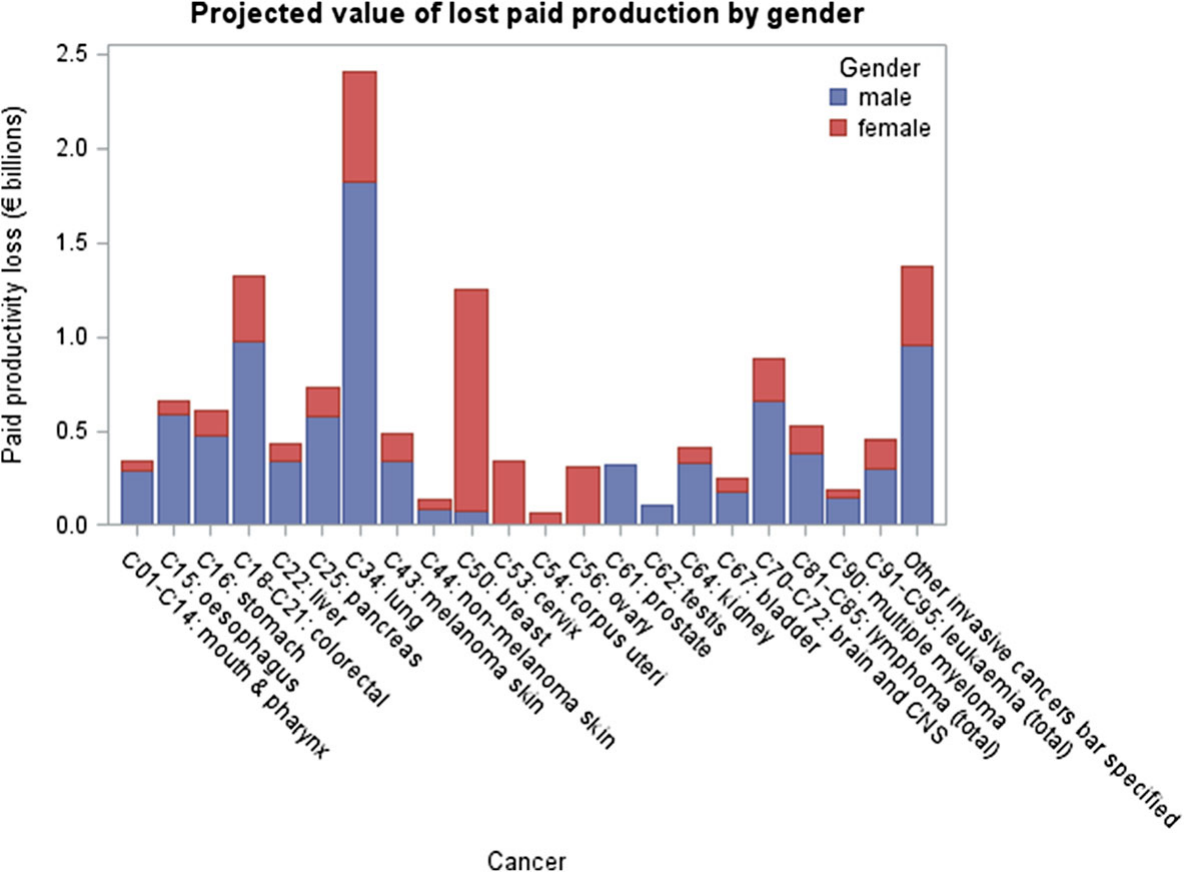
Projected value of lost paid production by gender

**Fig. 2:**
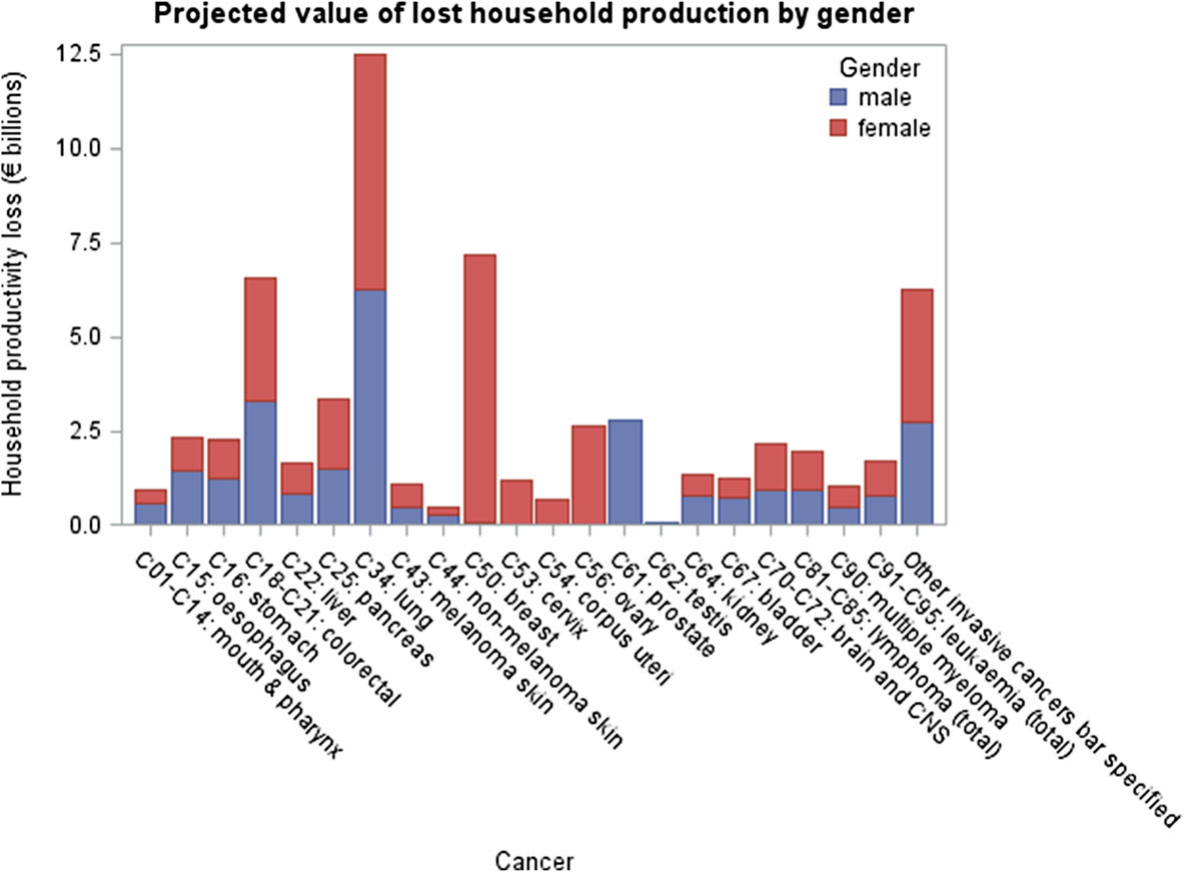
Projected value of lost household production by gender

Males account for 55 % of all invasive cancer deaths between 2011 and 2030; 66 % of the total lost paid production costs; and 42 % of the total lost household production. This reflects gender-specific patterns in labour force participation, wage rates, and household production. Figures 1 and 2 display the results for each cancer by gender, and the pattern of male dominated paid production and female dominated household production is seen across most sites.

**Fig. 3:**
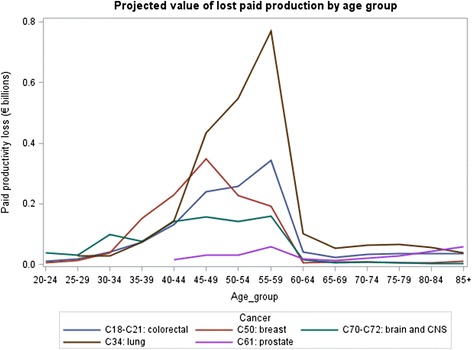
Projected value of lost paid production by age group

**Fig. 4:**
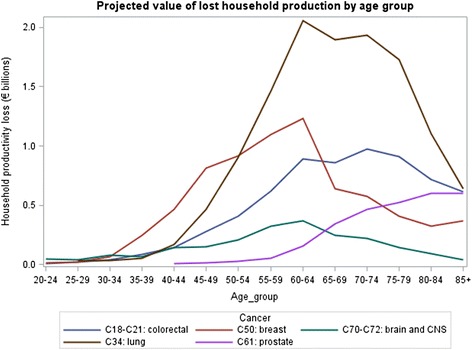
Projected value of lost household production by age group

Figures 3 and 4 displays lost production by age group for five cancers: breast, brain and central nervous system, colorectal, lung, and prostate. There is a large peak in the 55–59 age group, with a sharp decline after age 60.

### Sensitivity analyses

**Fig. 5:**
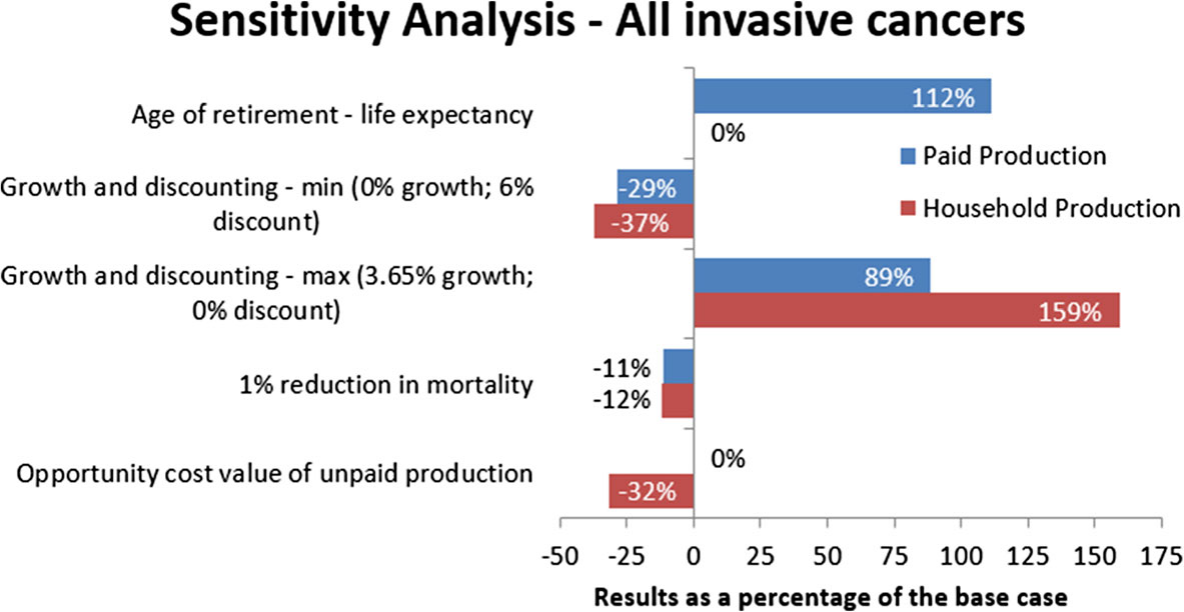
Sensitivity Analyses – All invasive cancer results for paid and household production as a percentage of the base case

The results of the sensitivity analyses are shown in Tables 2, 3 and Fig. 5. A 1 % reduction in deaths per 100,000 in all invasive cancers reduces total productivity loss to 88 % of the base case – a saving of €1.5 billion for paid production and €7 billion for household production (Table 2).

**Table 2:** Sensitivity analysis – reductions in mortality by 1 % per year for specified cancers

Cancer	Projected number of deaths	Value of lost paid production (million €)	Value of lost household production (million €)	Projected number of deaths (% of base case)	Value of lost paid production (million €)	Value of lost household production (million €)
	Base case			1 % Annual reduction in mortality		
C18-C21: colorectal	27,631	1321	6567	23,344 (84 %)	1169	5765
C25: pancreas	13,488	733	3355	11,673 (87 %)	649	2947
C34: lung	48,922	2403	12,493	41,730 (85 %)	2124	10,970
C50: breast	18,308	1255	7194	15,430 (84 %)	1113	6338
C70-C72: brain & CNS	6513	886	2164	5265 (81 %)	786	1907
C91-C95: leukaemia	6693	454	1691	5096 (76 %)	402	1486
*C00-C96: all invasive*	*233,168*	*12,685*	*60,427*	*206,336 (89 %)*	*11,231*	*53,086*

**Table 3:** Sensitivity analysis – using opportunity cost value of household production, instead of replacement cost

Cancer	Total lost household productivity (base case)	Total lost household productivity (opportunity cost)	% of base case
	(Million €)	(Million €)	%
C01-C14: mouth & pharynx	€954	€975	102
C15: oesophagus	€2322	€2067	89
C16: stomach	€2265	€1824	81
C18-C21: colorectal	€6567	€4571	70
C22: liver	€1682	€1330	79
C25: pancreas	€3355	€2392	71
C34: lung	€12,493	€8730	70
C43: melanoma skin	€1109	€1096	99
C44: non-melanoma skin	€492	€374	76
C50: breast	€7194	€3444	48
C53: cervix	€1218	€763	63
C54: corpus uteri	€706	€239	34
C56: ovary	€2626	€987	38
C61: prostate	€2794	€1911	68
C62: testis	€81	€175	217
C64: kidney	€1374	€1228	89
C67: bladder	€1240	€830	67
C70-C72: brain and CNS	€2164	€2129	98
C81-C85: lymphoma (total)	€1989	€1483	75
C90: multiple myeloma	€1032	€670	65
C91-C95: leukaemia (total)	€1691	€1267	75
Other invasive cancers bar specified	€6282	€4286	68
C00-C96: all invasive cancer deaths	€60,427	€41,286	68

Assuming that some individuals continue working past the effective retirement age results in a large increase in paid productivity loss. For all invasive cancers between 2011 and 2030 the value of lost paid production is estimated at €27 billion (212 % of the base case). Household production was not affected, as this was not influenced by retirement age in the base case.

Using age and gender specific wages to value household productivity resulted in a 32 % reduction in the estimate of lost household productivity between 2011 and 2030. However, this was not consistent between cancers, with corpus uteri reducing by 66 %, while testis cancer increased by 117 % (Table 3).

Varying the growth and discount rates results in a range of 71 % to 189 % (€9 to €24 billion) of the total paid production losses for all invasive cancers, and a range of 63 % to 259 % (€38 to €157 billion) of the total household productivity losses for all invasive cancers.

## Discussion

This analysis demonstrates the substantial productivity loss incurred by society due to cancer-related premature mortality - €73 billion in cumulative net present value in Ireland over the next 20 years. To put these losses into perspective, they represent approximately 1.4 % of Ireland’s gross domestic product (GDP; €159 billion) annually [23]. These projections can inform investment decisions at a time of aging populations, increasing cancer incidence and later retirement.

It is difficult to compare these results with previous estimates due to differing approaches and assumptions. Our results for 2012 are similar to or lower than previous single year estimates of total paid productivity losses due to cancer in Europe (including Ireland) [3, 6, 7]. This may be explained by our use of effective retirement age rather than pensionable age as a cut off for lost paid production, and our higher discount rate (5 % in our study compared to 3.5 % [6, 7] or 4 % [3] in other studies). In contrast, our estimates of productivity loss per cancer death are lower than seen in a US study of projected cancer mortality costs 2000 to 2020 [5], likely due to differences in settings such as the US having higher wage rates (14 % higher than Ireland [24]), later retirement and higher workforce participation rates [5].

Despite any differences in magnitude, the spread of lost productivity across different cancer sites in our results is very similar to those seen in previous studies [3, 5–7]. For paid productivity losses, cancers with high incidence in working age individuals, such as colorectal and lung cancer, are most costly overall. When cost per death is considered, cancers that occur in younger people, like testis, brain and cervical cancer, incur higher costs despite lower incidence because of the additional potential productive years of life lost. These two outcomes provide complementary perspectives for informing cancer control activities. When taken into consideration along with incidence and mortality, lost productivity estimates suggest that investment in a range of interventions, including some which target working age people, is important.

The results of this analysis can also be compared to cost of illness studies in other diseases in Ireland. The annual lost paid productivity due to premature mortality in this study is almost double that of both cardiovascular disease [25] and suicide [26], nearly seven times that of schizophrenia [27], and over 155 times that of dementia [28]. These results highlight the relative impact of cancer on the economy, and allow public policy to take a broader perspective on healthcare planning and prioritisation.

While higher paid productivity losses were seen for men within the analysis, these are due to higher wages and workforce participation rates in males. These differences are therefore driven by economic factors, rather than by value.

The inclusion of household production offers a counterbalance to estimations based on wage alone, which often undervalues contributions of women and younger and older workers who have reduced paid workforce participation. Household production is rarely included in estimates of disease burden in Europe [29], which results in an underestimation of the burden incurred by society. However, this study shows these losses are substantial – between three and five times the losses due to paid production, depending on the method of valuation (opportunity cost or replacement cost). Studies that include household production often do not give a breakdown of paid and household production [30–33]. A study in the US used a narrower definition of household production (including only housework and caring) and found household productivity losses to be approximately double those of paid production [5].

The sensitivity analysis demonstrates the impact of various assumptions within the analysis. Over the last 20 years cancer mortality has reduced in Ireland and worldwide by between 1 % and 2 % annually (averaged across all cancer types, age groups and genders) through improved screening and earlier detection, improved diet, improved treatments and reduced tobacco use [34]. The impact on productivity losses of continuing this trend provides a strong argument for economies to invest in strategies to reduce cancer mortality, as despite discounting within the calculations giving initial years more weight, the increasing population results in ongoing savings throughout the period.

In addition to improved treatments, an ongoing reduction in cancer mortality in Ireland may be achieved through other mechanisms [34]. Smoking rates in Ireland remain higher than in other countries, but are declining [35]. Given the number of cancers in which smoking is aetiologically implicated [36], and growing evidence of smoking as a prognostic factor [37], implementation of further initiatives to support smoking cessation (and reduce uptake) could yield significant economic benefits. National breast cancer screening is relatively new in Ireland, so the full benefits have not yet been realized [38]. A new screening programme for colorectal cancer has recently been introduced [39] and there is potential for spiral CT to be used for high risk lung cancer screening [40]. The HPV vaccine, provided to girls through a schools based programme, has high uptake in Ireland and should have a significant impact on cervical cancer rates in the future, particularly if extended to boys [41]. Finally, reducing inequalities in cancer diagnosis, management and prevention for those in lower socioeconomic groups, with lower education levels, or with comorbidities could significantly improve cancer mortality [34, 42].

The age of retirement makes a large difference in the estimates, with increasing workforce participation after retirement age increasing productivity loss by €14 billion. Ireland has a relatively high proportion of workers over retirement age, perhaps related to the high level of farming activity in the country. Given the potential for retirement ages to rise further in the future, the base case assumption are likely an underestimate of the true loss [22].

One of the unique aspects of this study is the projection of productivity losses into the future. This is particularly useful for diseases, such as cancer, where prevention is a key component of control. Typical assessments of disease burden discount avoidable costs in the future. Estimating future productivity loss draws specific attention to the importance of prevention, to inform the healthcare resource allocation debate.

While these future projections are important, they are also based on assumptions of the population in the future [13], which are inherently uncertain. In Ireland, migration is the most influential factor in determining population change, and is subject to significant fluctuations [13]. High migration together with substantial unemployment at present in Ireland means the Human Capital Approach may underestimate the future burden on society, however the message regarding relative, as well as the absolute, costs across cancers and activities remains important. Similarly, recent research suggests the increased levels of unemployment seen following the economic crisis in 2008 were associated with an increase in cancer mortality [43], which has not been accounted for in our mortality projections, and could mean our estimates provide a lower bound of the true magnitude of lost productivity due to cancer-related premature mortality.

The use of a 1 % mortality reduction across all cancers, rather than trends specific for certain cancer types is somewhat crude and there is an opportunity for future research to use detailed data to project changes in mortality for individual cancers. For example, screening programs have had clear impacts on the incidence (and possibly mortality) of breast, prostate and cervical cancers in Ireland [9]. The recent introduction of a colorectal cancer screening program, which includes people of working age in the target population [39], and the human papilloma virus vaccine targeting cervical cancer [44], could both result in reduced productivity loss into the future, on top of the typical measures of impact such as incidence.

A further limitation is that this analysis is limited to productivity losses resulting from premature mortality. Other forms of productivity loss, such as temporary workplace absence or early retirement, the costs associated with cancer detection and treatment, the costs to family members or the impact on quality of life are not captured, largely due to a lack of data. These all contribute to the financial toxicity of cancer, and the ongoing improvements in cancer mortality mean services such as financial planning and employment rehabilitation are increasingly essential components of treatment.

## Conclusion

Cancer mortality will result in €73 billion in productivity losses in Ireland from 2011 to 2030, 83 % of which is due to lost household production. These losses represent approximately 1.4 % of Ireland’s GDP annually. These results highlight that while reducing incidence and mortality of high incidence cancers is important, so too are interventions and policies to reduce lower incidence cancers, which disproportionately impact working age individuals. These estimates provide valuable evidence to policy makers regarding resource allocation decisions for cancer prevention and control.

## Abbreviations

CSO: Central Statistics Office
GDP: Gross domestic product
OECD: Organisation for Economic Cooperation and Development
US: United States of America
